# New hope for the survival of the Amur leopard in China

**DOI:** 10.1038/srep15475

**Published:** 2015-12-07

**Authors:** Guangshun Jiang, Jinzhe Qi, Guiming Wang, Quanhua Shi, Yury Darman, Mark Hebblewhite, Dale G. Miquelle, Zhilin Li, Xue Zhang, Jiayin Gu, Youde Chang, Minghai Zhang, Jianzhang Ma

**Affiliations:** 1 Feline Research Center of the Chinese State Forestry Administration; College of Wildlife Resources, Northeast Forestry University, Harbin 150040, China; 2 Department of Wildlife, Fisheries and Aquaculture, Mississippi State University, Mail Stop 9690, Mississippi State, MS 39762, USA; 3 WWF–China, Harbin 150040, China; 4 WWF–Russia, Amur Branch, Verkhneportovaya 18A, Vladivostok 690003, Russia; 5 College of Forestry and Conservation, University of Montana, Missoula, MT 59812, USA; 6 WCS, 2300 Southern Boulevard, Bronx, NY 10460, USA

## Abstract

Natural range loss limits the population growth of Asian big cats and may determine their survival. Over the past decade, we collected occurrence data of the critically endangered Amur leopard worldwide and developed a distribution model of the leopard’s historical range in northeastern China over the past decade. We were interested to explore how much current range area exists, learn what factors limit their spatial distribution, determine the population size and estimate the extent of potential habitat. Our results identify 48,252 km^2^ of current range and 21,173.7 km^2^ of suitable habitat patches and these patches may support 195.1 individuals. We found that prey presence drives leopard distribution, that leopard density exhibits a negative response to tiger occurrence and that the largest habitat patch connects with 5,200 km^2^of Russian current range. These insights provide a deeper understanding of the means by which endangered predators might be saved and survival prospects for the Amur leopard not only in China, but also through imperative conservation cooperation internationally.

Large carnivores exert substantial effects on the structure and function of diverse ecosystems^1^. Unfortunately, large carnivores are experiencing drastic declines in their populations and geographic ranges around the world^1^. For instance, the leopard (*Panthera pardus*), as a species, is near threatened and occupies only 65% of its historicalrange^2^. The Amur or Far Eastern leopard (*Panthera pardus orientalis* Schlegel 1857) is one of nine extant subspecies of leopard^3^. Historically, it was distributed broadly in the southernmost part of Russia’s Far East, northeastern China and much of the Korean Peninsula^4^. At the height of its power, the Amur leopard’s historic range reached 361,756 km^2^ worldwide but decreased to 71,971 km^2^ by the 1970s. Its current range is about 10,709 km^2^ in northeastern China and the Russian Far East, only 2.96% of its historical range (http://amur-heilong.net/Gis_site/gis_index.html). Since 1970, its reported population size has not been over 50 individuals in Russia^5^. In 1998, fewer than 10 individuals were found in China^6^. Furthermore, these populations were isolated and faced imminent extinction due to poaching, illegal harvesting of prey, habitat fragmentation and inbreeding depression^7^. Consequently, the Amur leopard may be the most rare felid worldwide and, in 1996, was the first listed as critically endangered on the IUCN red list^8^.

To recover the population of this rare big cat worldwide, experts argue that reintroduction may be the best option, despite serious pressure from human disturbance, and have conducted assessments for reintroduction preparation based on potential habitat and population size in the Russian Far East, using data on current and historicalrange^9^.

In China, over the past decade, Amur leopard conservation has focused on conducting transect line surveys, camera trap surveys, compensation for livestock preyed upon by wildlife and other recordings. There were 8 ~ 11 Amur leopards found during a local survey in the southern Laoyeling Mountains in Jilin during the winter of 2011–2012^10^, and an additional 5 ~ 7 leopards identified in another local survey in the southern Laoyeling Mountains of Heilongjiang during the winter survey of 2012–2013 (http://www.tx2.org.cn/News/ShowArticle.asp?ArticleID=894). Since the spring of 2012, Chinese experts have undertaken camera trap surveys about this elusive animal across the parts of the Hunchun–Wangqing region of Jilin province. Subsequently, the first breeding evidence of a female wild Amur leopard with two kittens was obtained in October 2013 by camera traps in northeastern China^11^. Since 1998, measures for natural forest protection, nature reserve construction projects and bans on wildlife hunting and forest harvesting are in place in northeastern China. These measures ensure that the structure and quality of forest habitat substantially improve, aiding in wildlife survival. However, the status of the current range of the Amur leopard, new areas for potential habitat, as well as actual and potential population sizes, are still unclear. Also, little is known about factors that determine Amur leopard distribution and limit its survival. In this study, we aim to explore how much current range area exists in northeastern China, what factors limit the leopard’s spatial distribution and determine the population size and the extent of potential habitat.

## Occurrence evidence for Amur leopard, Amur tiger and prey

**Figure 1:**
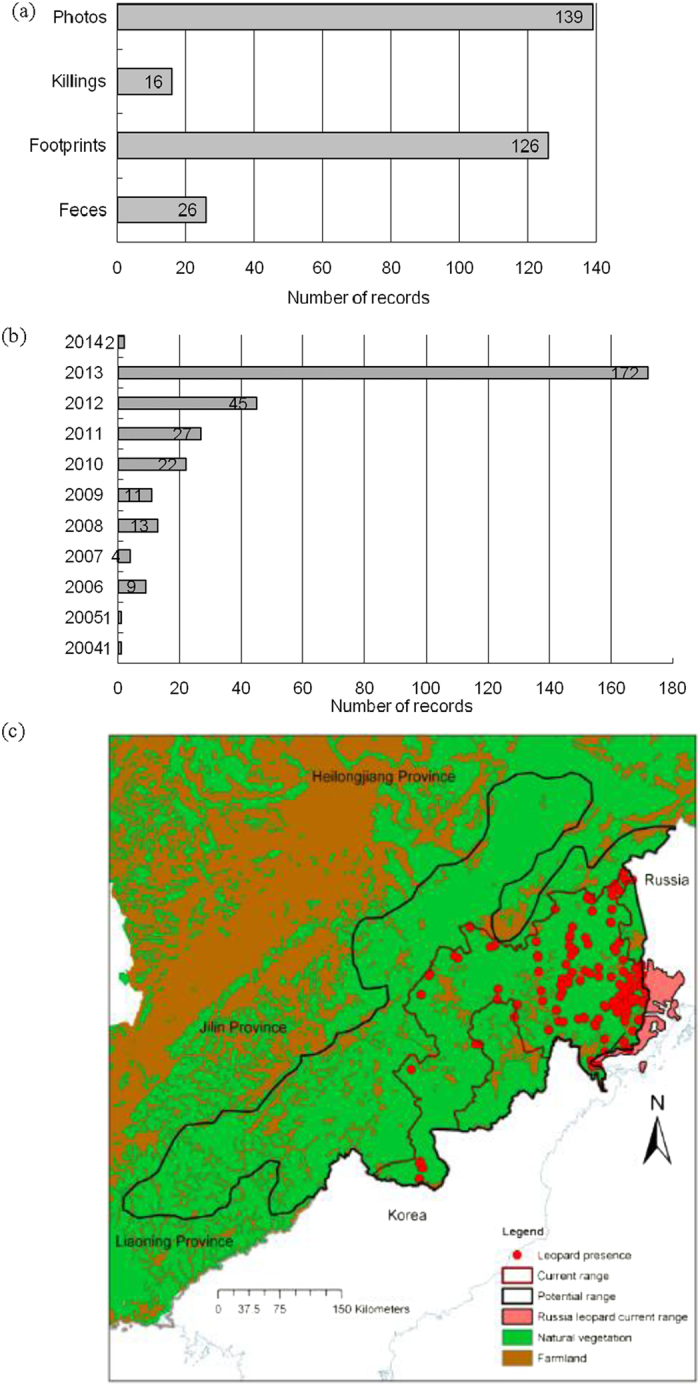
Occurrence information type (**a**) and time (**b**) for Amur leopard confirmed and (**c**) illustration of the distribution of occurrence information points, current and historical range of Amur leopard in northeastern China. The pink color represents the current range of the Amur leopard in Russia Far East. Maps were created using ArcGIS software by Esri (Environmental Systems Resource Institute, ArcGIS 10.0; www.esri.com).

Within the historical range of the Amur leopard in China (Amur Heilong Database, http://amur-heilong.net/Gis_site/gis_index.html), during field work, we recorded 307 occurrences of Amur leopards during 2004–2014 (Fig. 1a,b). Prey presence was recorded in 1,190 200-m segments, including 780 segments for roe deer, 76 for red deer, 131 for sika deer and 203 for wild boar, along survey routes with a total length of 894.8 km during the four winters of 2010–2014 (Extended Data Figs 1–5). A total of 384 occurrences of Amur tigers were recorded during 2004–2014 (Extended Data Fig. 6). The leopard occurred in a current range of 48,252 km^2^ in an area within its historical range of 137,950 km^2^ in northeastern China. The current range in northeast China is connected with the 5,200 km^2^ current range of the leopard in Russia (Fig. 1c).

**Figure 2:**
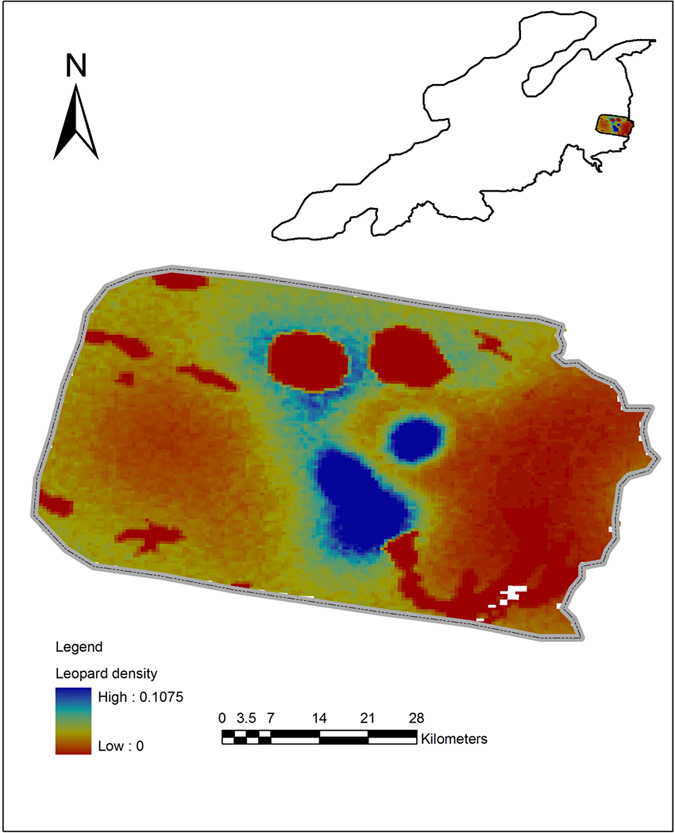
Amur leopard density distribution predicated by Spatially Explicit Capture Recapture Model (SECR). The color gradient of each pixel represents the density gradient from red (low density) to blue (high density) of Amur leopard population at each pixel. Only pixels judged to be suitable habitat are included and the size of each pixel is 1 km^2^. Maps were created using ArcGIS software by Esri (Environmental Systems Resource Institute, ArcGIS 10.0; www.esri.com).

Based on camera trap surveys, 10 individual leopards were unambiguously identified by the program Extract Compare from 68 photographic captures over about a 12-month period (476 trap nights) in an area of 1,214 km^2^, suggesting that leopard density is 0 ~ 0.107 individuals per 1 km^2^ in this survey area (Fig. 2). The 95% credible interval (CI) of the total number of leopard individuals was 10 ~ 24 individuals based on Bayesian spatial capture–recapture model parameters (Extended Data Table 2)^12^. Furthermore, we not only found the Amur leopard breeding family (See Video s1)^11^, but on 4 November 2014, in the same camera trap survey area, two sub-adults living with a female Amur tiger were also found (See Videos 2).

## Amur leopard, Amur tiger and prey occurrence model

**Figure 3:**
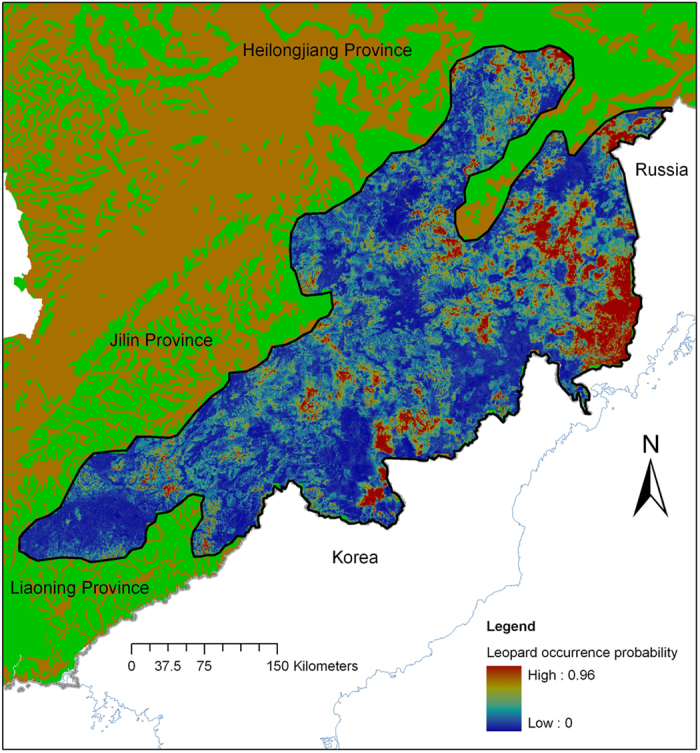
Spatial distributions showing occurrence probabilities for Amur leopard in northeastern China, as predicted using distribution modeling. Maps were created using ArcGIS software by Esri (Environmental Systems Resource Institute, ArcGIS 10.0 (www.esri.com).

Presence of the leopard, tiger and four ungulates, i.e., roe deer (*Capreolus pygargus*), red deer (*Cervus elaphus*), sika deer (*Cervus nippon*) and wild boar (*Sus scrofa*), as individual species and combined, were determined at different scales and used to obtain their habitat suitability based on bias files correction and habitat factors^13^. We found the models had different predication abilities at different scales. However, using training and test data we found that the Amur leopard model has maximum of sum of AUC values at the 400 m scale (Extended Data Fig. 7), so we obtained occurrence probability layers of Amur tiger and prey at this scale (Extended Data Fig. 8–13), determining suitable habitat areas by cutoff points based on the maximum sum of model sensitivity and specificity (Extended Data Table 3)^14^. In addition, we revealed factors that drive the distribution of the tiger and the various prey species (Extended Data Table 4). We found that human disturbance, temperature and vegetation played crucial roles in ungulate species distribution and that human disturbance and prey base drove Amur tiger distribution (Extended Data Table 4, Extended Data Fig. 14). Considering a combination of habitat factors, Amur tiger and prey occurrence probability, we obtained information concerning Amur leopard occurrence probability (Fig. 3) and key habitat factors (Table 1), all of which demonstrated that prey distribution was the most important factor driving Amur leopard distribution (Table 1 and Extended Data Fig. 15).

**Figure 4:**
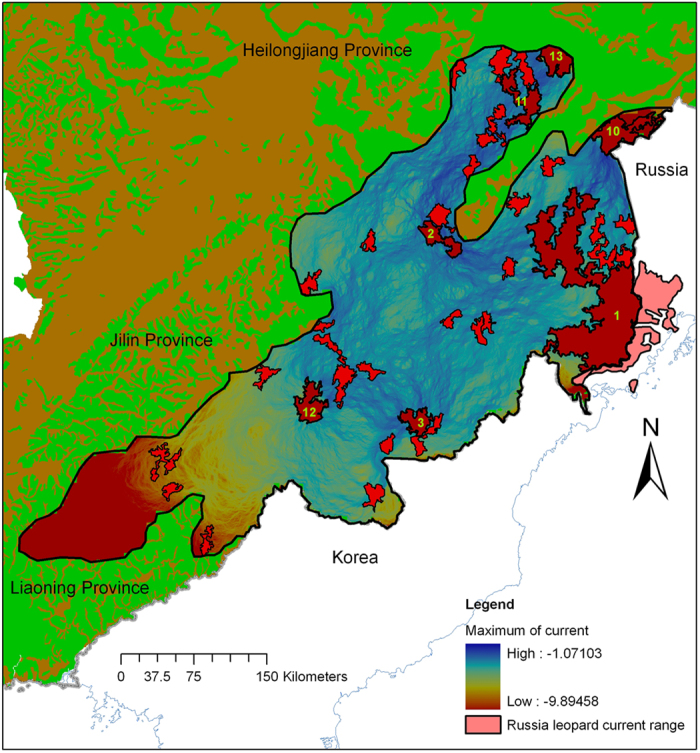
Habitat connectivity map among the suitable habitat patches based on the Circuitscape 4 Software analysis. Yellow numbers identify the big suitable patches >500 km^2^ derived from the distribution model.

Based on the Amur leopard occurrence probability layer, we determined suitable patches >500 km^2^ (i.e., large patch) and >100 km^2^ in current and potential regions and assessed connectivity among the 7 large patches (i.e., 3 patches are in the leopard’s current range and 4 in its potential range) (Fig. 4). We suggest that good connectivity happened among suitable large patches and most small patches existed in important corridors as stepping-stones, except for several small patches in the southwestern part of the study area (Fig. 4). Furthermore, we found that largest suitable patch in the current region is connected with Russian habitat patches and may be an important source site for Amur leopard recovery in these two countries.

## Potential population assessment and its relation to connectivity

**Figure 5:**
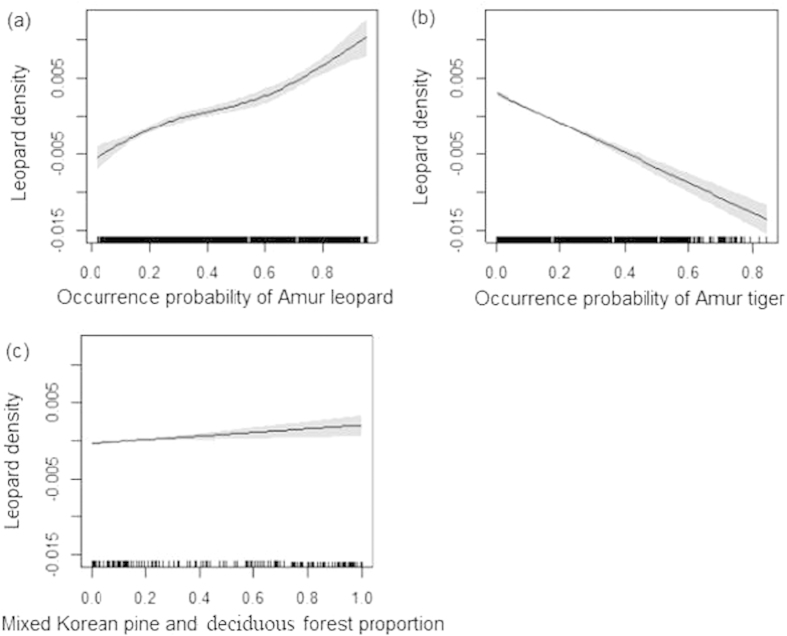
Partial probability response curves of Generalized Additive Models (GAMs) for the Amur leopard region of northeastern China based on occurrence probability of Amur leopard, occurrence probability of Amur tiger and mixed Korean pine-deciduous forest proportion. The *x*-axis is the value of the model independent variable and the y-axis is the additive contribution of the variable to the non-parametric GAM smoothing function. Shaded areas are two standard errors about the estimated function.

Using Generalized Additive Models (GAMs), we found the Amur leopard density distribution was strongly positively related to both Amur leopard occurrence probability and mixed Korean pine and deciduous forest proportion and negatively related to occurrence probability of the Amur tiger (Fig. 5), showing that the Amur leopard preferred a highly suitable habitat with a high proportion of mixed Korean pine and deciduous forest where they could avoid their competitor.

Based on the best fitting GAM of Amur leopard density (Fig. 5), we predicted the population size of potential suitable patches in current and potential ranges (Table 2). Our results showed that approximately 195.1 (136.4 ~ 253.5) individuals exist in the total 21,173.7 km^2^ area of 37 suitable patches in current and potential habitat patches. We found that approximately 100.7 (59.1 ~ 142.1) individuals exist in 11,292 km^2^ of suitable patches in the current range and the largest patch (i.e., Laoyeling across the Jilin and Heilongjiang provinces) with an area of 8,625 km^2^, neighboring the current leopard range in Russia, may harbor 72.5 (36.1 ~ 108.8) individuals (Table 2).

We found that improved connectivity between habitat patches would likely support higher leopard density (Extended Data Fig. 16), suggesting that corridor quality of surrounding suitable patches plays a crucial role in elevating carrying capacity of the patches for the Amur leopard.

## Discussion

The historical range of the leopard was greater than that of any other of the larger carnivores, since it inhabited the whole of Asia and was found almost throughout Africa. The Amur Heilong Database shows that the Amur leopard only occupies 2.96% of its historical range and most experts predict that there is no hope of natural recovery for the subspecies because of its very small population size, limited available habitat and restricted present range^7^,^9^. However, we found that the present population size, available habitat and present range are all greater than what was previously understood. In this study, we found 48,252 km^2^ of current range for the Amur leopard in China and, therefore, the current range of the Amur leopard internationally may encompass more than 53,000 km^2^, including 5,200 km^2^ of current range in the Russian Far East. Furthermore, based on the camera trap data model, we predicted that potential population size in its current range may be over 100 individuals, 10 times higher than the 1998 estimate^6^, and about 195 individuals may be supported in the 21,173.7 km^2^ of potential suitable patches in northeastern China. What is more, we found that the largest suitable habitat patch (8,625 km^2^) in China may harbor over 72 individuals that are interconnected with the current leopard distribution range in Russia. In October 2013, using camera traps in this patch, we also recorded a breeding Amur leopard female with two kittens^11^. Consequently, this patch crossing the Sino-Russia border area may play a key role as the Amur leopard population core area.

Good quality information on the spatial distribution of critically endangered species is very important for determining conservation and monitoring prioritization tasks. Such spatial information is also critical to decision-makers and managers, so that forest resources are sustainably used and the negative impacts of human activities are mitigated in key places. The largest patch, taken together with contiguous habitat in Russia, may support 120 individual leopards and a population of that size is crucial to the maintenance of genetic diversity and to the avoidance of inbreeding depression. Nevertheless, populations of approximately this size located in one patch may still face the risk of extinction^15^. Accordingly, international cooperation between Russia and China is urgently needed to jointly manage the critically endangered species core area conservation at a landscape scale. Both countries should consider identifying areas among suitable patches at which to maintain the perviousness of the border to prompt population migration and elevate occupancy capacity of habitat patches^9^.

Carbone and Gittleman (2002) suggested that 10,000 kilograms of prey supports about 90 kilograms of a given carnivore species, there fore the 0.5 to 37.04 per 100 km^2^ Amur leopard population density requires 300 to 416,300 kg per 100 km^2^ of prey biomass distribution^16^. The leopard relies on small- to medium-sized ungulate prey in both summer and winter^17^. During winter, the Amur leopard diet mainly consists of small- to medium-sized deer and smaller young wild boar^17^. This shows that leopard populations are related not only to prey biomass, but also to prey size^18^. Although some prey species are not main dietary components, they may compensate for reductions in some other species. Thus, we considered four ungulate prey species as vitally important to Amur leopards. In addition, snow track data are closely related to absolute density, and occurrence and abundance are also related to this^19^. Inclusion of prediction probability of the above prey models in the leopard model could be interpreted as suitable areas for leopard selection^9^. Hence, prey distribution may be critical to the development of a stable Amur leopard population as it is a fundamental determinant of leopard density. Our findings indicated that occurrence probability of prey is an extremely important driver of Amur leopard distribution, especially roe deer, which are preferred by the Amur leopard^17^,^20^. According to research^17^,^20^, leopard diet largely depended on roe deer (up to 66%), wild boars (up to 8%), Siberian musk deer (up to 9%), sika deer (up to 6%), as well as other species, which served as prey for the Amur leopard. Amur tiger preyed mainly wild boar (up to 43%) and roe deer (up to 9%), red deer (up to 78.7) and other species. In addition, the Amur leopard or tiger may change dietary components by prey compensation resulting from prey reduction or prey availibility^17^,^20^. However, in northeast China, the two dominant ungulate species, i.e., wild boar and roe deer, may simultaneously drive both Amur leopard and tiger distribution. In China, roe deer may play a more important role for the existence of the current range of the critically endangered Amur leopard^17^. However, the roe deer has not been listed as an important protected species in China and is often hunted by local people; thus, as staple food of the Amur leopards, roe deer population conservation should be a management priority, especially due to its relationship to the current and potential suitable range of the Amur leopard.

Our study suggested that the presence of railways and temperature are also key habitat factors influencing the distribution of most ungulate species. We do not have evidence of the direct effects of railways on ungulates but the existence of railways does lead to increased human activity (including firewood collection, non-timber forest product harvesting, grazing in forests, illegal hunting and so on), which have been shown to negatively affect ungulates both directly and indirectly through diminished habitat quality^21^ and thus affects the distribution of their predator, the Amur tiger^22^. In addition, in northeastern China, cold winters are often accompanied with deep snow and snow may increase the death rate of ungulates, negatively affecting ungulate survival and reproduction during this harsh season^23^. Hence, when a snowstorm occurs in China or Russia, managers should consider rescue measures for ungulates by providing supplementary food in priority areas for big cat conservation. Villages and roads similarly influenced prey distribution, which may pose an increased risk of direct poaching or indirect habitat loss and fragmentation for ungulates and their predators^24^. Accordingly, to elevate the quality of Amur leopard habitat, the first task is to change the behavior of local people by encouraging them to adopt sustainable rural livelihood measures and minimize negative effects on Amur leopard prey, to improve habitat quality of the prey^25^. Thus, guaranteeing an abundant prey base may induce more opportunities to spread out from source sites, prompting the Amur leopard to move into a larger range.

Leopards and larger felids appear to coexist through niche the partitioning of ungulate prey based on body size^26^. Therefore, in places where Amur leopards and tigers coexist, conservation should focus on prey assembly, not only on prey population abundance, but also on prey species diversity. Otherwise, the Amur leopard may be in an inferior position and its population size may decrease, distribution range may shrink and food items may shift to livestock, all of which would lead to more leopard-human conflicts with a corresponding tiger population increase^27^. During the Amur leopard survey in the Russia Far East, it was unfortunate that two cases of Amur leopards chased and killed by Amur tigers were found. These cases provide a mechanism to explain our finding that leopard density is inversely related to tiger occurrence probability. Consequently, for the conservation of the critically endangered Amur leopard, the impacts of intraguild competition of sympatric carnivores may be an important limiting factor and this is a factor that is rarely considered while planning endangered top predator guild recovery programs^27^. Although we found an inverse relationship between leopard density and tiger occurrence probability, the finding of an example of sympatric leopard and tiger families indicates that the direct relationship between the two species is complex and coexistence appears to rely on temporal separation, likely maintained by the leopard. After the Russia St. Petersburg Declaration during the International Tiger Forum (i.e., the “Tiger Summit” held 21–24 November 2010), on 29 July 2011, China issued the Wild Tiger Recovery Action Plan. To establish a migration corridor near the Sino-Russia border area, the three connected national-level nature reserves (i.e., the Hunchun, Wangqing and Laoyeling natural reserves) were constructed across international and inter-provincial border areas. Furthermore, the Global Environmental Fund Project focusing on the Amur tiger and their habitat conservation at the landscape leve lis being conducted in northeastern China. This project will cost US $ 18millionfor three years and will run from 2014 to 2017. The Amur tiger is a priority target for conservation; its population has shown an increasing trend and distribution range has increased rapidly during recent years. For example, during 2014, the Amur tiger re-occurred in four counties (i.e., Fuyuan, Linkou, Huanan and Fangzheng counties of Heilongjiang province), where there has been no tiger occurrence for more than two decades. Because of the increase in the Amur tiger population in northeastern China, managers should monitor the population trends of abundance and distribution dynamics of the Amur leopard in a timely manner and, they should adopt comprehensive conservation measures by considering prey community structure mediation, habitat landscape and vegetation quality improvement, as well as anthropogenic disturbance control. What is more, based on this study, both suitable habitat patches and potential population size should prompt the application of conservation practices and managers should monitor these measures effectively. Effective conservation will benefit the entire Amur leopard population across China, Russia and, in the future, even in the Korea Peninsula. Consequently, this study reveals recovery prospects for the critically endangered Amur leopard in China and provides a basis for understanding important information involving intraguild impacts of sympatric predators, prey distribution effects, anthropogenic drivers and habitat landscape connectivity.

After the “Tiger Summit”, both the Chinese and Russian governments began to conduct joint conservation projects with the aim of doubling the Amur tiger population. It is expected that this will bring about many benefits for the Amur leopard and greatly extend results from tiger habitat conservation projects. More attention should be paid to the Amur leopard because of their much smaller population size and their imminent threat of extinction. Their conservation is also more complex due to factors such as being a sympatric large predator with the Amur tiger; however, it is urgent to make a grassroots Amur Leopard Conservation Action Plan, according to Amur leopard habitat landscape requirements. In 2014, the Ministry of Natural Resources and the Environment of the Russian Federation issued the “Strategy for Conservation of the Amur Leopard in the Russian Federation” pointed out that Russian Amur leopard conservation needed to strengthen international cooperation with the Chinese side and that focus should be on studying the interaction and competitive relationships between the Amur leopard and Amur tiger. If these guidelines are realized, recovery of the critically endangered Amur leopard worldwide may be achieved, especially due to China’s prioritizing of the crucial local responsibility as stewards of such a large potential Amur leopard population and habitat.

## Methods

### Amur leopard, tiger and ungulate presence data collection

First, we mapped the historical range of the Amur leopard in the Changbaishan Mountains in northeastern China referring to Amur Heilong Database (http://amur-heilong.net/Gis_site/gis_index.html). Then we collected Amur leopard presence data from particular Amur leopard route surveys. From January 2004 to January 2014, in the leopard’s historical range, locations of occurrence were obtained from camera trap surveys, records of compensation for livestock predation and patrolling records^10^. We confirmed the occurrence of both the Amur leopard and the Amur tiger by photographic evidence, snow or mud footprints with front pad characteristics, kill sites and DNA extraction from fecal or hair samples.

We collected ungulate snow track data from the Amur leopard route survey over 4 winters (2010–2011, 2011–2012, 2012–2013 and 2013–2014). The survey route was designed with a density of 36 km/100 km^2^ and located in Amur leopard and tiger habitat locations. The total length of survey routes was 894.8 km and was located in the Amur leopard current region of northeastern China. We divided the survey route into 200 m segments in order to count the presence of four ungulate species known to form the prey base for both the leopard and tiger populations in this region: roe deer (*Capreolus pygargus*), red deer (*Cervus elaphus*), sika deer (*Cervus nippon*) and wild boar (*Sus scrofa*) by distinguishing different characteristics of their snow tracks. Leopards prey on a variety of species but they rely on small- to medium-sized ungulates, whereas tigers prefer larger prey than the leopard^17^. Considering the total biomass of prey required to meet the dietary requirements of both the leopard and the tiger, we combined the potential distribution of these four prey species as an important factor influencing the presence of Amur leopards.

### Landscape and biotic covariates

Species distribution modeling considers characteristic scales of habitat factors associated with different levels of organization of a species^28^. We adopted a combination of climate, vegetation, anthropogenic influences, topographical features and river covariates in order to understand the Amur leopard, tiger and their prey distribution (Extended Data Table 1). Temperature, snow depth, Normalized Difference Vegetation Index (NDVI) and elevation data were derived using Moderate-resolution Imaging Spectroradiometer (MODIS)(Shuttle Radar Topography Mission [SRTM])^29^. Vegetation types, villages, roads, rivers and railway vector data were obtained from the Environmental and Ecological Science Data Center for West China, National Natural Science Foundation of China. Vegetation was classified mainly as typical of northeastern China, i.e., farmland, shrub, oak forests, birch forests, deciduous and mixed conifer-deciduous forests, larch forests, mixed Korean pine-deciduous forests and spruce-fir forests^9^. Using ArcGIS 9.3 Spatial Analyst, all landscape habitat layer data were re-sampled as different scale data from 200 m, 400 m, 800 m, 1,600 m, 3,200 m, 6,400 m, 12,800 m and 25,600 m in order to select the best model for predicting Amur leopard and Amur tiger distribution and also prey availability^30^.

For factors affecting prey and predator (i.e., Amur tiger), we first selected the best model for each of the four and all prey species at a spatial scale, then probability of occurrence was used for Amur tiger modeling selection. Both prey and tiger occurrence probability layers derived from models were used as landscape variables for building Amur leopard distribution models (Extended Data Table 1)^31^.

### Model development and potential suitable habitat patch identification

We used the number of presence pixels for each of the four ungulate species, total prey, Amur leopard and tiger presence on each of the eight spatial scales, as independent variables. Using R software, we tested the multicollinearity among the same scalar characteristic habitat predictor variables based on a combination of variance inflation factors and Spearman’s rank correlation (*rs *< 0.5). The mostly uncorrelated predictor variables were used as initial input to the models. Maximum entropy is widely used to model species geographic distributions (i.e., their occurrence), using only record occurrence localities. We used the software Maxent (Version 3.3.3 k)^32^ to build distribution models for prey, the Amur tiger and then the Amur leopard with the subset of predator environment variables.

To deal with areas that were without records after our survey efforts, we felt we could not discriminate between areas that where unsuitable patches from those that where under-sampled and so we used weighted presence data and background samples from current and potential distribution areas as described in the paper of Elith *et al*. (2010)^13^. The weights by extrapolation were used as a bias grid in Maxent to improve the reliability of models^13^.

We used *k*-fold cross-validation and the area under the receiver operating characteristic (ROC) area under curve (AUC) to evaluate the predictive ability of models. Models with AUC value 0.7 to 0.9 were regarded as useful and >0.9 as highly accurate^33^. To determine a suitable spatial scale for modeling, we selected models at the scales with maximum AUC values (i.e., plus AUC values of both the training and test data) as final models^34^. To identify suitable habitat patches in the forest mosaic, we determined cut points in estimated habitat suitability (i.e., occurrence probability values) using ROC curves. We identified cutoff points where the sum of model sensitivity and specificity was maximized^14^, as this method should obtain higher prediction success for rare species^35^. To determine suitable patches, we converted suitable pixels into polygon data and then used ArcGIS 9.3 Spatial Analyst to connect suitable pixels and form the closed edge of suitable patches, establishing delimitated suitable habitat patches in both the current and historical regions. Then, we calculated the area of each suitable patch and counted the number in the area >100 km^2^ or >500 km^2^ using ArcGIS 9.3^9^.

### Camera trap survey and estimating potential leopard population size

We conducted camera trap surveys of the Amur leopard population in a 1,214.53 km^2^ area of Jilin province based on the Amur leopard information from route survey in northeastern China from April 2013 to July 2014. First, we divided the Amur leopard survey area into units, each approximately 10 km^2^. Camera traps were located on animal trails or where traces were found within each unit and 2 cameras were set at each point, opposite each another, in order to increase capture probability and also to capture the pattern on both side of each leopard^36^. Cameras were attached to trees, 45 ~ 50 cm above the trail, and at a distance of 3.5 ~ 4 m from the expected trajectory of the animals, to maximize the quality of the images^37^. The average distance between camera traps was 3.7 km (min = 1.4 km; max = 4.1 km). This distance is suitable given that female Amur leopard home ranges are estimated as being between 45 ~ 65 km^2^^38^. A total of 76 camera traps were used for a total of 476 trap nights. Camera trap data were collected once every 2 months. For the camera trap data, we used ExtractCompare software to identify photos of Amur leopards, considering the location and time they were captured^36^. Then, we used SPACECAP, a user-friendly software package in R, to estimate animal densities using spatially explicit capture recapture models based on camera trap data^12^. Spatial capture–recapture models not only substantially dealt with problems posed by individual heterogeneity in capture probabilities in conventional capture–recapture analyses, but also offered a way to estimate spatial animal density distribution based on a unified Bayesian modeling framework^39^.

We chose Generalized Additive Models (GAMs as implemented in the R package mgcv), a flexible and nonparametric method for calibrating species response to environmental predictors^40^. To build a leopard population density prediction model based on a random selection for 15% of pixel datasets (i.e., to avoid sample pseudo-replication^41^) in camera trap areas, all habitat variables, together with tiger occurrence probability, leopard probability and prey occurrence probability were derived from Maxent models in this camera trap area and were examined by Spearman’s rank correlation (*rs *< 0.5) and then the best-fitting models from the candidate models were selected by following the rule of minimization of generalized cross validation (GCV) on the condition that all variables must be statistically significant (*P *< 0.05)^42^. Finally, we use the best fitting GAM for Amur leopard spatial population density prediction to calculate the leopard density of each pixel of each suitable patch identified by Maxent in R package mgcv. We obtained the mean and standard deviation of density in each suitable patch and assessed the total number of individuals in all suitable patches in ArcGIS 9.3.

### Patch connectivity and its relation to leopard density distribution

Considering the significance of the large patch (>500 km^2^) as current or future source sites, we used occurrence probability derived from the distribution model combined with connectivity analysis of Circuitscape software based on circuit theory to identify potential corridors for movement of leopards among these patches. Circuit theory complements commonly used connectivity models because of its connections to random walk theory and its ability to simultaneously evaluate contributions of multiple dispersal pathways^43^. We calculated relative resistance of movements between habitat patches assuming that resistance value was inversely related to the probability of occurrence from the Maxent model, according to the method of Chetkiewicz and Boyce (2009)^44^. Thus, we used the inverse of the probability of occurrence as the resistance function in Circuitscape 4.0.1 to determine the most connected leopard patches within >500 km^2^ and identify movement corridors.

We buffered suitable big patches by a distance of 10 km^45^ and then calculated mean connectivity values within the buffer zone of each patch based on the connectivity map derived from Circuitscape 4.0.1 in ArcGIS 9.3. To explore whether connectivity around a patch affects its population density distribution or not, we used a linear model to detect the relationship between the mean surrounding connectivity value and leopard density predicted of patches.

All study was in accordance with the guidelines approved by The American Society of Mammalogists^46^. Our camera trapping protocol was assessed and approved by Expert Committee of Feline Research Center of Chinese State Forestry Administration.

## Additional Information

**How to cite this article**: Jiang, G. *et al*. New hope for the survival of the Amur leopard in China. *Sci. Rep*. **5**, 15475; doi: 10.1038/srep15475 (2015).

## Supplementary Material

Supplementary Tables

Supplementary Figures

## Figures and Tables

**Table 1:** Relative contributions of each predictor variable to the Amur leopard distribution model.

Predictor variable	Contribution (%)	Permutation importance
Occurrence probability of prey	50.0	56.1
Snow depth	15.7	19.5
Spruce-fir forest proportion	15.6	0.7
Distance to road	9.2	8.5
NDVI	4.2	8.6
Distance to village	3.3	5.4
Mixed Korean pine-deciduous forest proportion	1.9	1.2
Total predictor variables	100	100

**Table 2:** 

Current Patch No.	Patch name	Area (km^2^)	Mean density (Ind./km^2^)	Population size	95% C.I.
1	Laoyeling	8625	0.008	72.5	36.1–108.8
2	Ningan–Dongjingcheng	600	0.011	6.6	5.1–8.0
3	Baihe–Helong	558	0.011	6.3	5.1–7.3
4	Suiyang	387	0.009	3.5	2.9–4.0
5	Changbai (a)	330	0.011	3.9	3.0–4.7
6	Dongning–Suiyang	246	0.009	2.3	1.9–2.6
7	Tianqiaoling–Wangqing	198	0.010	2.1	1.7–2.4
8	Changbai (b)	180	0.010	1.9	1.6–2.0
9	Wangqing–Yanji–Longjing	168	0.009	1.7	1.2–2.0
	Total habitat patches	11292	0.010	100.7	59.1–142.1
**Potential patch No.**					
10	Jidong	1154.1	0.0089	10.3	7.8–12.7
11	Hailin–Linkou	918.2	0.0101	9.2	7.5–11
12	Jinyu–Fusong	809.3	0.0097	7.9	6.6–9
13	Linkou–Boli	683.0	0.0091	6.2	4.9–7.5
14	Huadian(a)	472.6	0.0102	4.8	4–5.7
15	Linkou(a)	433.6	0.0094	4.1	3.4–4.7
16	LinkouB	361.1	0.0094	3.4	2.8–4
17	Tonghua–Xinbin–Qingyuan	413.4	0.0078	3.2	2.5–3.9
18	Helong	346.9	0.0109	3.8	3.1–4.5
19	Ningan–Hailin	356.8	0.0110	3.9	3.2–4.6
20	Ningan–Mudanjiang	289.0	0.0103	3.0	2.5–3.4
21	Hailin(a)	303.0	0.0098	3.0	2.4–3.5
22	Jiaohe	269.4	0.0099	2.7	2.1–3.2
23	Huadian (b)	255.8	0.0103	2.6	2.2–3.1
24	Muleng–Linkou	261.3	0.0103	2.7	2.2–3.1
25	Dongning(a)	233.0	0.0070	1.6	1.1–2.1
26	Dongning (b)	220.0	0.0089	1.9	1.5–2.4
27	Panshi–Huinan–Huadian	240.2	0.0090	2.2	1.9–2.4
28	Fangzheng–Yanshou	192.0	0.0090	1.7	1.4–2.0
29	Longjing	217.1	0.0095	2.1	1.7–2.4
30	Dunhua	206.4	0.0097	2.0	1.8–2.2
31	Hailin (b)	192.6	0.0122	2.3	2.1–2.6
32	Hailin(c)	194.9	0.0103	2.0	1.7–2.3
33	Tonghua–Xinbin	179.4	0.0078	1.4	1–1.8
34	Jian	203.4	0.0060	1.2	0.8–1.6
35	Antu	167.2	0.0107	1.8	1.4–2.1
36	Dunhua–Wuchang–Jiaohe	185.1	0.0116	2.1	1.8–2.4
37	Huadian–Jiaohe	123.2	0.0091	1.1	0.9–1.2
	Total potential habitat	9882.1	0.0095	94.4	77.3–111.4
Total		21173.7		195.1	136.4–253.5

Habitat-based population estimates for the 9 patches of Amur leopard habitat within their current range, 28 patches of Amur leopard habitat within their potential range in northeastern China based on Amur leopard population density predication of generalized additive model (GAM) developed in the parts of Hunchun–Wangqing region with camera trap data collected from April 2013 to July 2014. Patch name, area and predicted population size (with 95% credible interval [CI]) are shown for each ofthe 37 habitat patches.
