# Supplementary material for: New hope for the survival of the Amur leopard in China
Source: Sci Rep. 2015 Dec 7;5:15475. doi: 10.1038/srep15475 (PMC4670984; doi:10.1038/srep15475)
Supplement: Supplementary Tables [file srep15475-s1.docx]

**New hope for the survival of the Amur leopard in China**

Guangshun Jiang^1*^, Jinzhe Qi^1^, Guiming Wang^2^, Quanhua Shi^1,3^, Yury Darman^4^, Mark Hebblewhite^5^, Dale G. Miquelle^6^, Zhilin Li^1^, Xue Zhang^1^, Jiayin Gu^1^, Youde Chang^3^, Minghai Zhang^1^, Jianzhang Ma^1^

^1^Feline Research Center of the Chinese State Forestry Administration; College of Wildlife Resources, Northeast Forestry University, Harbin 150040, China. ^2^Department of Wildlife, Fisheries and Aquaculture, Mississippi State University, Mail Stop 9690, Mississippi State, MS 39762, USA. ^3^WWF–China, Harbin 150040, China. ^4^ WWF–Russia, Amur Branch, Verkhneportovaya 18A, Vladivostok 690003, Russia. ^5^College of Forestry and Conservation, University of Montana, Missoula, MT 59812, USA. ^6^WCS, 2300 Southern Boulevard, Bronx, NY 10460, USA.

^*^Corresponding author email: [jgshun@126.com](mailto:jgshun@126.com)

**Appendix:**

**Extended Data Table 1**｜**Habitat variables tested for association with Amur leopard, tiger and ungulate presence in northeastern China.** Data were processed in ArcGIS 10.0 in grid format (different pixel scales with resolutions of 200 m, 400 m, 800 m, 1,600 m, 3,200 m, 6,400 m, 12,800 m, and 25,600 m) based on re-sampled or interpolated measurements.

**Extended Data Table 2**｜**Posterior summaries of spatial capture-recapture model parameters for the Amur leopard camera trap data based on *N* = 10 observed individuals.** *N* is the number of Amur leopard activity centers in the population exposed to sampling and *D* is the density per 100 km^2^, ψ is the data augmentation parameters, and σ is the parameter in the bivariate normal pdf, β is regression coefficient that measures the behavioral response, and p0 is detection probability, the estimates of "p1" and "p2" are also reported. p1 is the encounter probability for individuals that have not previously been encountered, and p2 is the encounter probability for individuals subsequent to their initial encounter.

**Extended Data Table 3**｜**Suitable and unsuitable habitat areas, cutoff point of probability determined by species distribution models based on the 400 m pixel.**

**Extended Data Table 4**｜**Relative contributions of each predictor variable to the roe deer, red deer, sika deer, wild boar, total prey and Amur tiger distribution model in Amur leopard range of northeastern China.**

**Extended Data Figure 1**｜**Distribution of presence points of roe deer in current and historical ranges of the Amur leopard in northeastern China.** Maps were created using ArcGIS software by Esri (Environmental Systems Resource Institute, ArcGIS 10.0; [www.esri.com](http://www.esri.com)).

**Extended Data Figure 2**｜**Distribution of presence points of red deer in current and historical ranges of the Amur leopard in northeastern China.** Maps were created using ArcGIS software by Esri (Environmental Systems Resource Institute, ArcGIS 10.0; [www.esri.com](http://www.esri.com)).

**Extended Data Figure 3**｜**Distribution of presence points of sika deer in current and historical ranges of the Amur leopard in northeastern China.** Maps were created using ArcGIS software by Esri (Environmental Systems Resource Institute, ArcGIS 10.0; [www.esri.com](http://www.esri.com)).

**Extended Data Figure 4**｜**Distribution of presence points of wild boar in current and historical ranges of the Amur leopard in northeastern China.** Maps were created using ArcGIS software by Esri (Environmental Systems Resource Institute, ArcGIS 10.0; [www.esri.com](http://www.esri.com)).

**Extended Data Figure 5**｜**Distribution of presence points of total prey in current and historical ranges of the Amur leopard in northeastern China.** Maps were created using ArcGIS software by Esri (Environmental Systems Resource Institute, ArcGIS 10.0; [www.esri.com](http://www.esri.com)).

**Extended Data Figure 6**｜**Distribution of presence points of Amur tiger in current and historical ranges of the Amur leopard in northeastern China.** Maps were created using ArcGIS software by Esri (Environmental Systems Resource Institute, ArcGIS 10.0; [www.esri.com](http://www.esri.com)).

**Extended Data Figure 7**｜**AUC (Area Under the Curve) values of different scales for train and test datasets based on the Amur leopard Maxent models.**

**Extended Data Figure 8**｜**Spatial distribution showing occurrence probabilities for roe deer in current and historical ranges of the Amur leopard in northeastern China, as predicted using distribution modelling.** Maps were created using ArcGIS software by Esri (Environmental Systems Resource Institute, ArcGIS 10.0; [www.esri.com](http://www.esri.com)).

**Extended Data Figure 9**｜**Spatial distributions showing occurrence probabilities for red deer in current and historical ranges of the Amur leopard in northeastern China, as predicted using distribution modelling.** Maps were created using ArcGISH software by Esri (Environmental Systems Resource Institute, ArcGIS 10.0; [www.esri.com](http://www.esri.com)).

**Extended Data Figure 10**｜**Spatial distributions showing occurrence probabilities for sika deer in current and historical ranges of the Amur leopard in northeastern China, as predicted using distribution modelling.** Maps were created using ArcGISH software by Esri (Environmental Systems Resource Institute, ArcGIS 10.0; [www.esri.com](http://www.esri.com)).

**Extended Data Figure 11**｜**Spatial distributions showing occurrence probabilities for wild boar in current and historical ranges of the Amur leopard in northeastern China, as predicted using distribution modelling.** Maps were created using ArcGIS software by Esri (Environmental Systems Resource Institute, ArcGIS 10.0; [www.esri.com](http://www.esri.com)).

**Extended Data Figure 12**｜**Spatial distributions showing occurrence probabilities for total prey in current and historical ranges of the Amur leopard in northeastern China, as predicted using distribution modelling.** Maps were created using ArcGIS software by Esri (Environmental Systems Resource Institute, ArcGIS 10.0; [www.esri.com](http://www.esri.com)).

**Extended Data Figure 13**｜**Spatial distributions showing occurrence probabilities for the Amur tiger in current and historical ranges of the Amur leopard in northeastern China, as predicted using distribution modelling.** Maps were created using ArcGIS software by Esri (Environmental Systems Resource Institute, ArcGIS 10.0; [www.esri.com](http://www.esri.com)).

**Extended Data Figure 14**｜**Each of the curves represents a distribution model created using only the corresponding variable for roe deer, red deer, sika deer, wild boar, total prey and Amur tiger.**

**Extended Data Figure 15**｜**Each of the curves represents a distribution model created using only the corresponding variable for the Amur leopard.**

**Extended Data Figure 16**｜**Amur leopard density (individuals/km^2^) of each suitable patch responded to habitat connectivity of areas within a 10 km buffer zone of each suitable patch.** Amur leopard population density was predicated from the Generalized Additive Model (GAM) model and habitat connectivity value for y-axis has been transformed into Exp(y).

**Video legends:**

**Video 1**｜**Breeding Amur leopard video evidence of camera traps.**

**Video 2**｜**Breeding Amur tiger video evidence of camera traps.**

Extended Data Table 1

| Habitat factor | Description of the habitat factor | Data type | Unit |
| --- | --- | --- | --- |
| Climate |  |  |  |
| Snow depth | Mean snow depth of March, 2004-2014 year (source grid has 5 km resolution). | Continuous | (ratio) |
| Temperature | Mean night temperature of January, 2004-2010 year (source grid has 1 km resolution). | Continuous | (c) |
| Vegetation |  |  |  |
| NDVI | Mean NDVI value of September, 2004-2010 year (source grid has 500 m resolution). | Continuous | (ratio) |
| Forest type | Forest area, including all 8 forest types, calculated in each pixel. The vegetation vector map of China (1 : 1 million) in 2000 year was converted into grid with 200 m resolution. | Continuous | (km^2^) |
| Anthropogenetic factors |  |  |  |
| Distance to railway | Distance from the central point of each pixel to the railway. | Continuous | (km) |
| Distance to village | Distance from the central point of each pixel to village. | Continuous | (km) |
| Distance to road | Distance from the central point of each pixel to road. | Continuous | (km) |
| Topography and river |  |  |  |
| Elevation | Elevation grid with 90 m resolution. | Continuous | (m) |
| Elevation SD | Standard deviation of elevation of each pixel. Source elevation grid with 90 m resolution. | Continuous | (m) |
| Slope | Slope grid with 90 m resolution derived from the digital elevation model above. | Continuous | (m) |
| Slope SD | Standard deviation of slope grid with 90 m resolution derived from the digital elevation model above. | Continuous | (m) |
| Aspect | Aspect grid with 90 m resolution derived from the digital elevation model above. | Continuous | (m) |
| Distance to river | Distance from the central point of each pixel to river. The river includes the primary river and their branches. | Continuous | (km) |

Extended Data Table 2

| Parameter | Mean | SD | 5% | 95% |
| --- | --- | --- | --- | --- |
| ψ | 5.758 | 1.161 | 3.377 | 7.845 |
| σ | 0.002 | 0.002 | 0.001 | 0.007 |
| β | 1.615 | 0.498 | 0.475 | 2.433 |
| p0 | 0.338 | 0.100 | 0.160 | 0.540 |
| N | 16.575 | 3.989 | 10 | 24 |
| D | 0.620 | 0.149 | 0.374 | 0.897 |
| p1 | 0.002 | 0.002 | 0.001 | 0.007 |
| p2 | 0.770 | 0.150 | 0.422 | 0.927 |

Extended Data Table 3

| Species | Cutoff point | Number of pixels | Unsuitable habitat(km^2^) | Number of pixels | Suitable habitat(km^2^) | Training data (AUC) | Test data  (AUC) |
| --- | --- | --- | --- | --- | --- | --- | --- |
| Roe deer | 0.212 | 754138 | 120662 | 91232 | 14597 | 0.926 | 0.909 |
| Red deer | 0.034 | 754138 | 120662 | 91232 | 14597 | 0.974 | 0.934 |
| Sika deer | 0.066 | 767756 | 122841 | 88674 | 14188 | 0.977 | 0.965 |
| Wild boar | 0.141 | 721674 | 115468 | 134057 | 21449 | 0.945 | 0.894 |
| Total prey | 0.160 | 711683 | 113869 | 144048 | 23048 | 0.907 | 0.887 |
| Amur tiger | 0.170 | 800467 | 128075 | 55264 | 8842 | 0.922 | 0.897 |
| Amur leopard | 0.183 | 639809 | 102369 | 204686 | 32750 | 0.801 | 0.865 |

Extended Data Table 4

| Roe deer | Contribution (%) | Permutation importance |
| --- | --- | --- |
| Distance to railway | 40.8 | 25.5 |
| Temperature | 25.7 | 31.6 |
| Elevation | 15.5 | 20.6 |
| NDVI | 8.3 | 4.6 |
| Snow depth | 1.7 | 3.6 |
| Total predictor variables | 92 | 85.9 |

| Red deer |  |  |
| --- | --- | --- |
| Distance to railway | 63.1 | 56 |
| Distance to village | 10.2 | 5.5 |
| Temperature | 7.9 | 8.4 |
| Elevation | 5.6 | 14.3 |
| Oak forest proportion | 2.9 | 0.1 |
| Distance to road | 1.2 | 2.2 |
| Total predictor variables | 90.9 | 86.5 |
| Sika deer |  |  |
| Temperature | 34.7 | 29.2 |
| Distance to railway | 34.2 | 34.3 |
| Elevation | 11.5 | 11.1 |
| Distance to river | 4 | 3.2 |
| NDVI | 3.8 | 0.9 |
| Distance to road | 2.1 | 3.8 |
| Total predictor variables | 90.3 | 82.5 |
| Wild boar |  |  |
| Distance to railway | 44.2 | 31.4 |
| Elevation | 16.3 | 22.8 |
| NDVI | 12.2 | 2.8 |
| Temperature | 7.2 | 6.9 |
| Distance to road | 3 | 7.7 |
| Snow depth | 2.7 | 2.7 |
| Distance to village | 2.5 | 5 |
| Oak forest proportion | 2.1 | 4.2 |
| Total predictor variables | 90.2 | 83.5 |
| Prey |  |  |
| Distance to railway | 43.6 | 32.5 |
| Temperature | 23.3 | 22.8 |
| Elevation | 15.1 | 20.9 |
| NDVI | 9.3 | 6.1 |
| Total predictor variables | 91.3 | 82.3 |
| Amur tiger |  |  |
| Distance to railway | 49.6 | 38.2 |
| Occurrence probability of prey | 22.9 | 13.7 |
| Elevation | 14.3 | 8.4 |
| NDVI | 3.0 | 0.5 |
| Oak forest proportion | 1.9 | 10.5 |
| Distance to village | 1.3 | 3.2 |
| Snow depth | 1.2 | 1.0 |
| Total predictor variables | 94.2 | 75.5 |
