# Supplementary figures and images for: New hope for the survival of the Amur leopard in China
Source: Sci Rep. 2015 Dec 7;5:15475. doi: 10.1038/srep15475 (PMC4670984; doi:10.1038/srep15475)

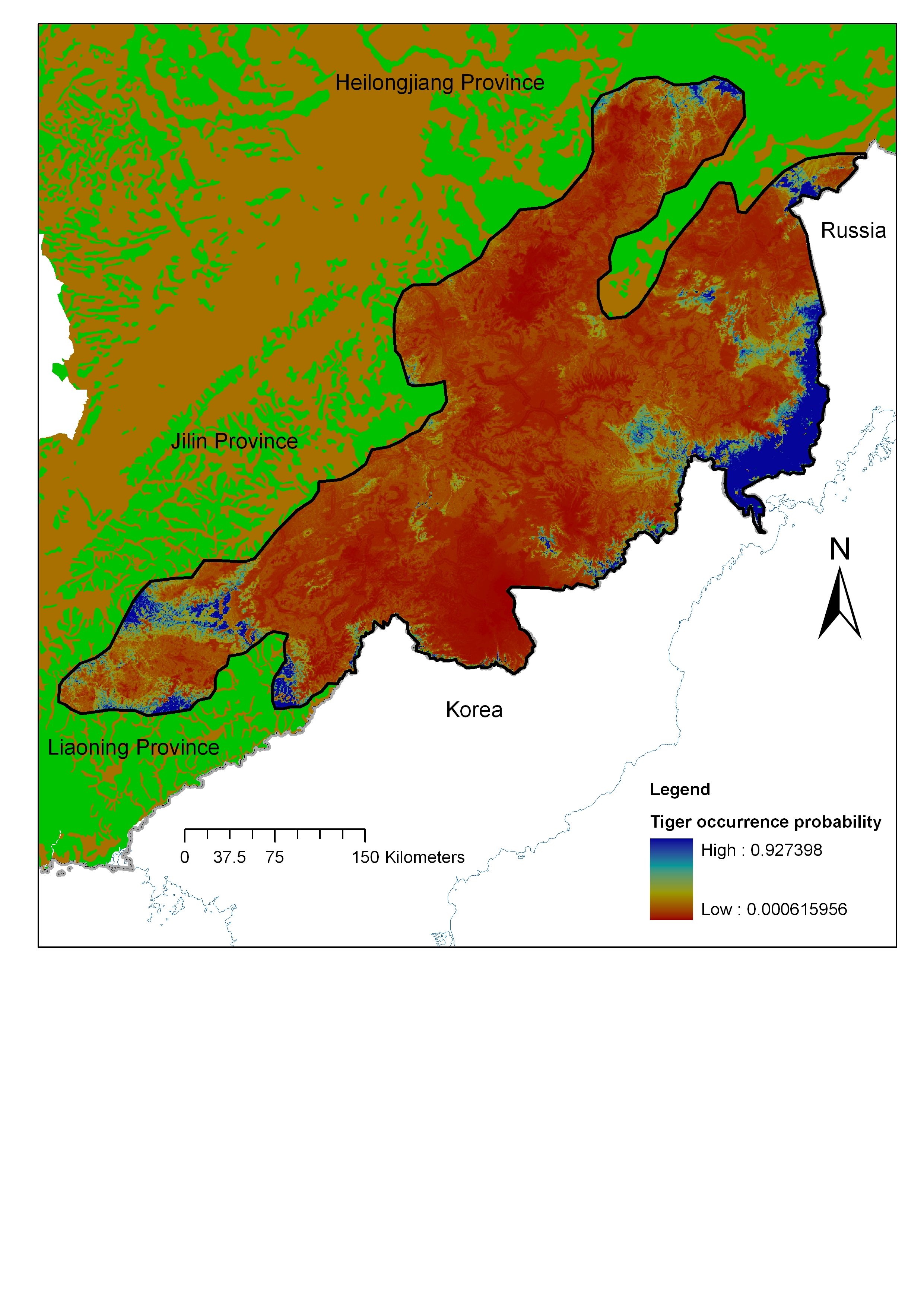

Supplement: Supplementary Figures [file srep15475-s2.zip › Extend data Fig. 13 Amur tiger occurrence probability.jpg]

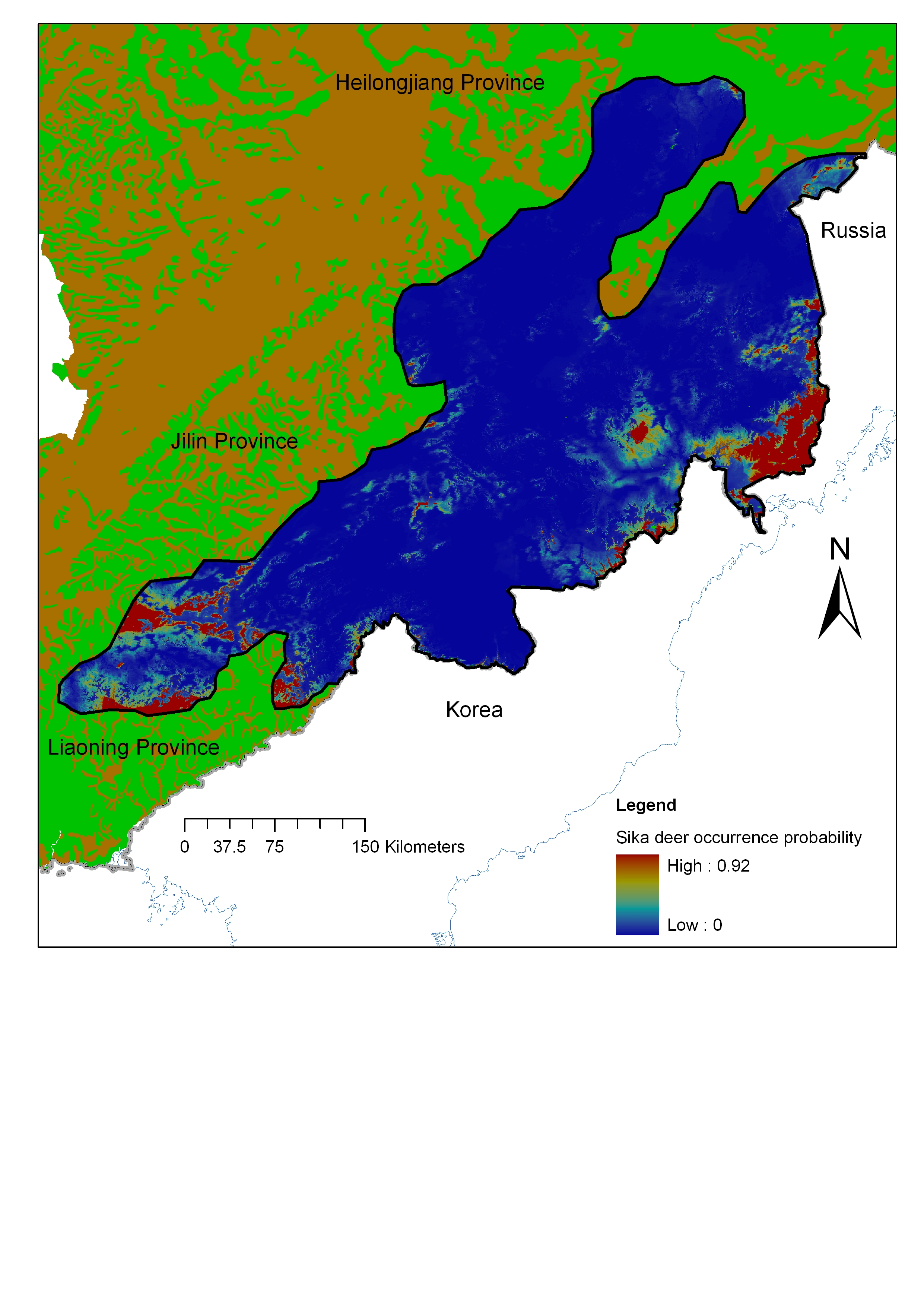

Supplement: Supplementary Figures [file srep15475-s2.zip › Extended data Fig. 10 Sika deer occurrence probability.jpg]

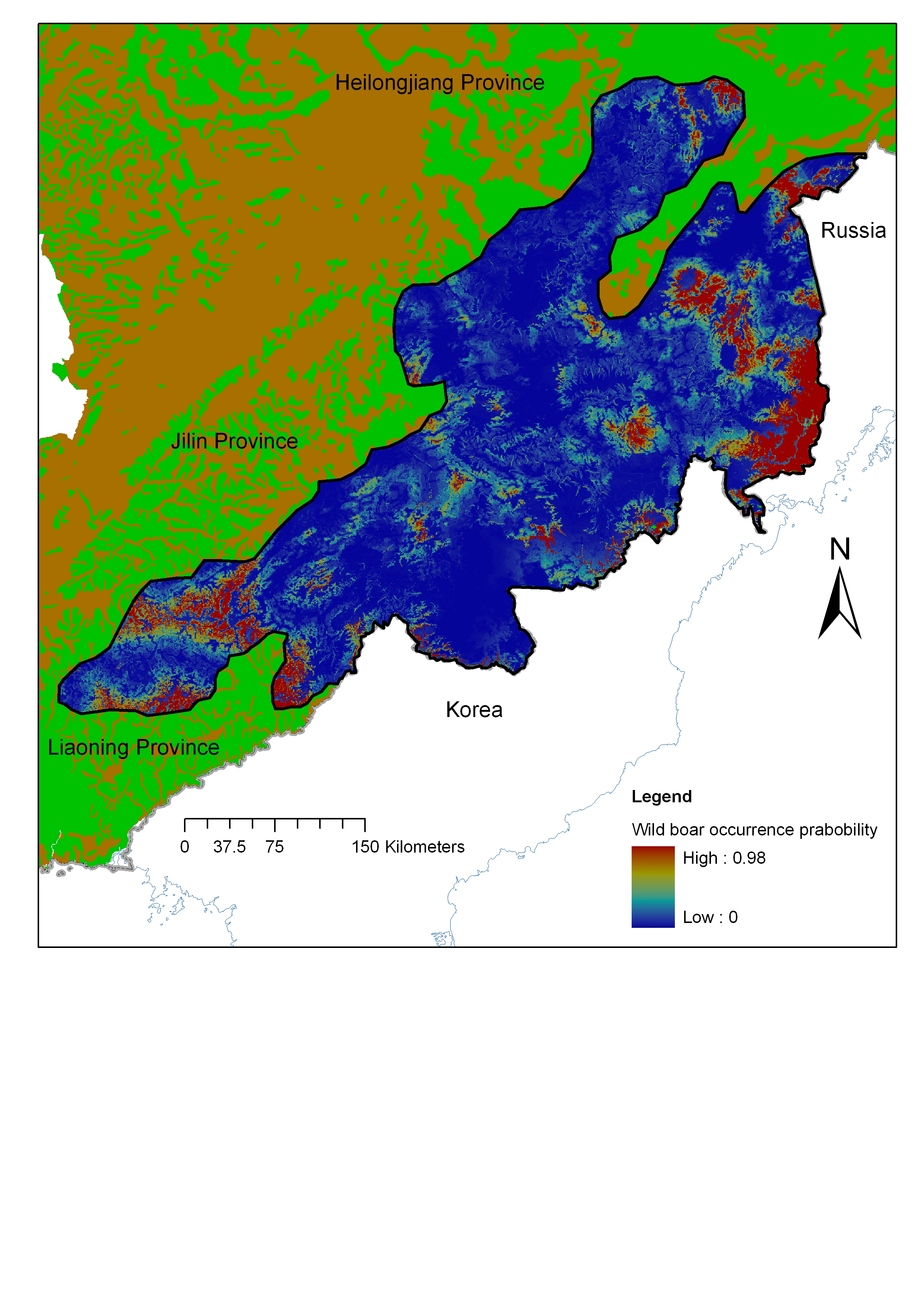

Supplement: Supplementary Figures [file srep15475-s2.zip › Extended data Fig. 11 Wild boar occurrence probability.jpg]

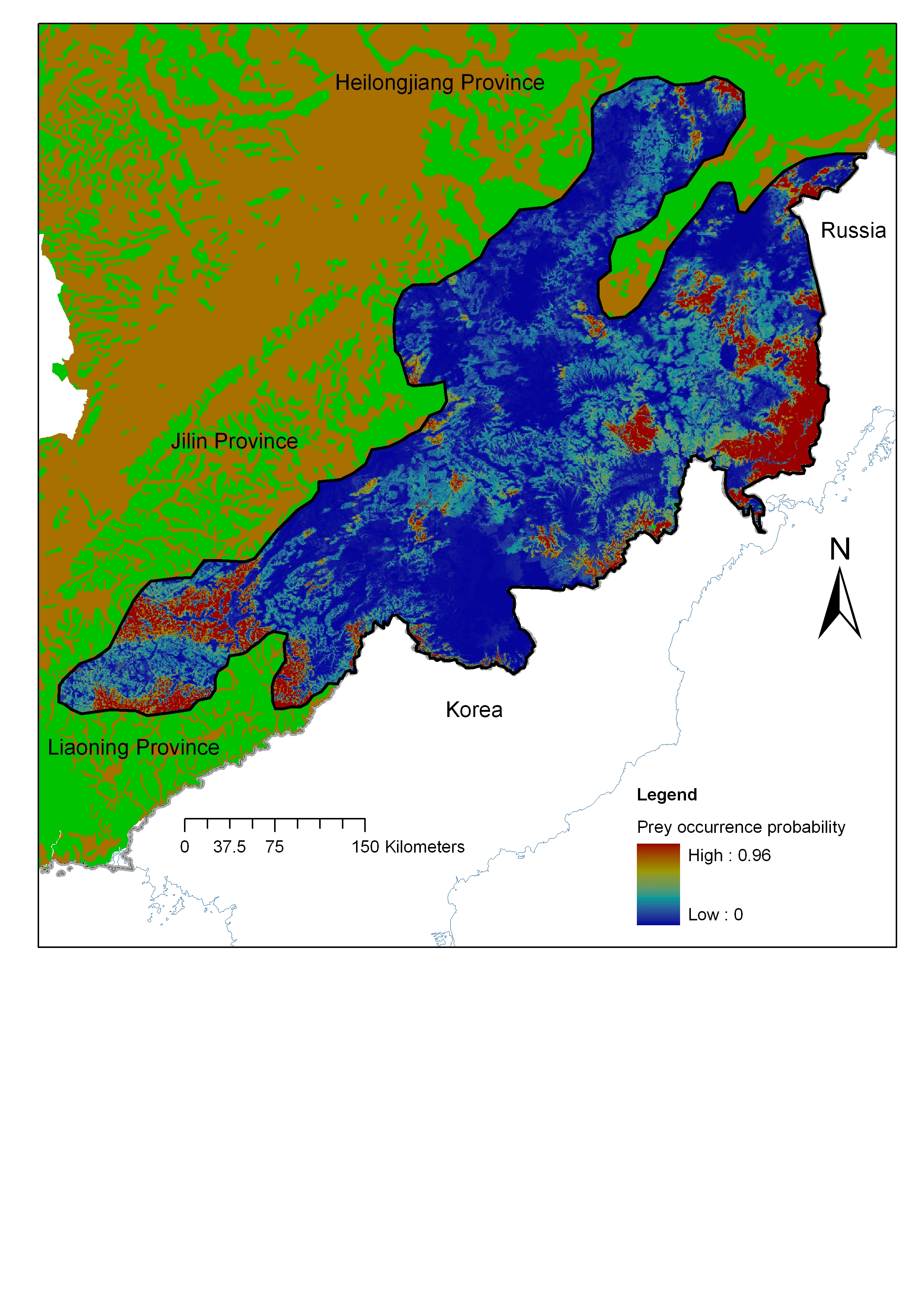

Supplement: Supplementary Figures [file srep15475-s2.zip › Extended data Fig. 12 Prey occurrence probability.jpg]

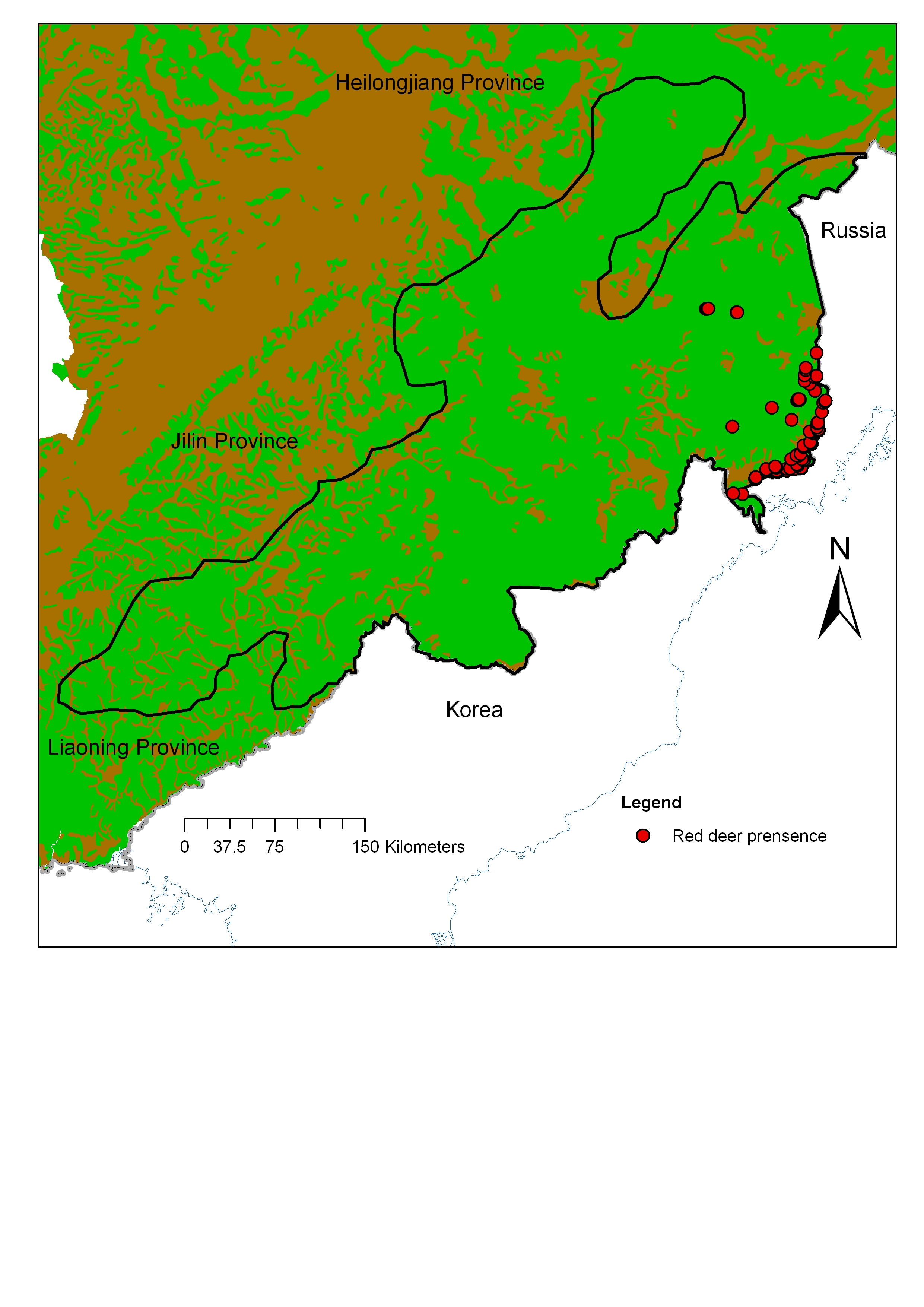

Supplement: Supplementary Figures [file srep15475-s2.zip › Extended data Fig. 2 Red deer presece.jpg]

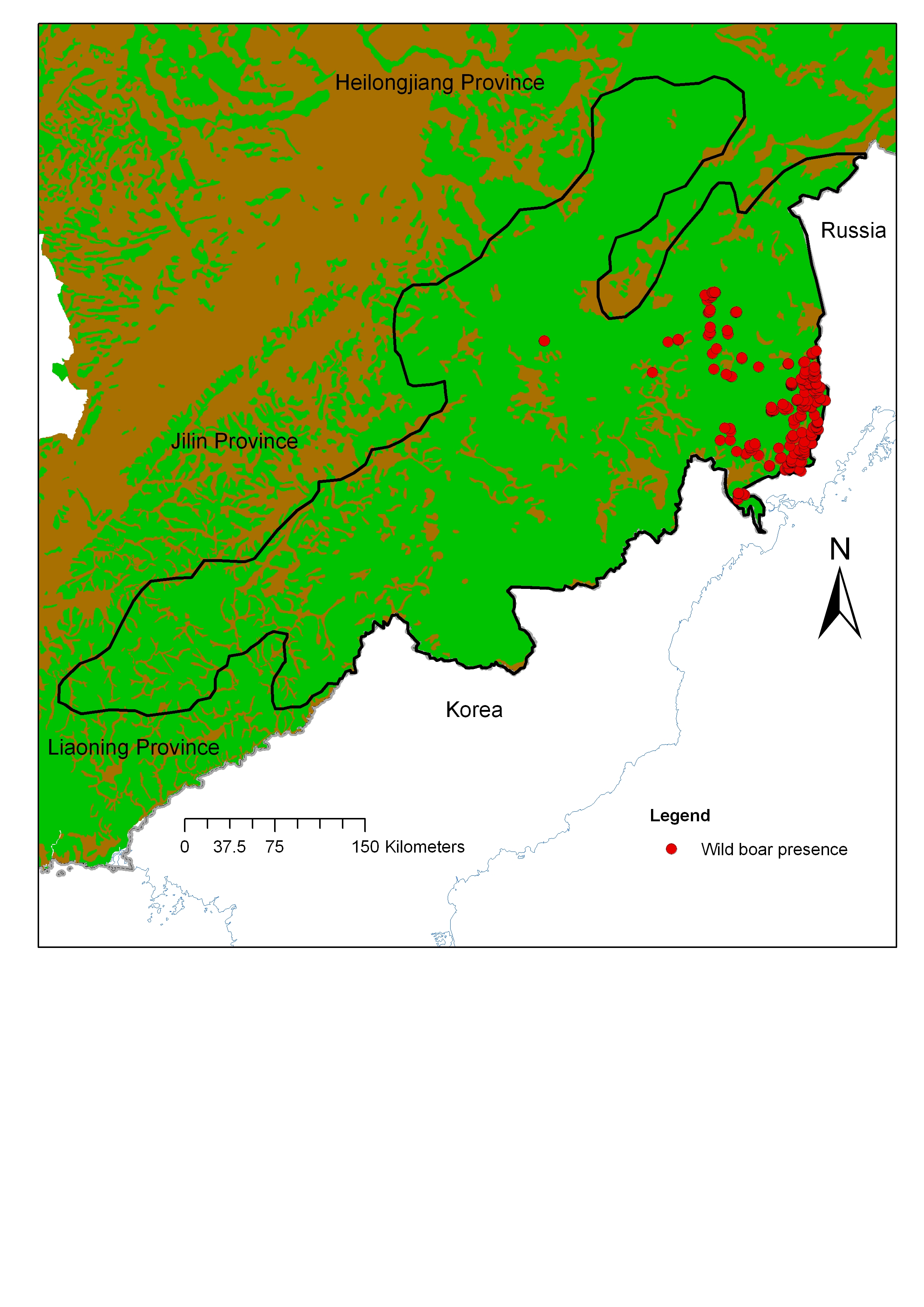

Supplement: Supplementary Figures [file srep15475-s2.zip › Extended data Fig. 4 Wild boar presence.jpg]

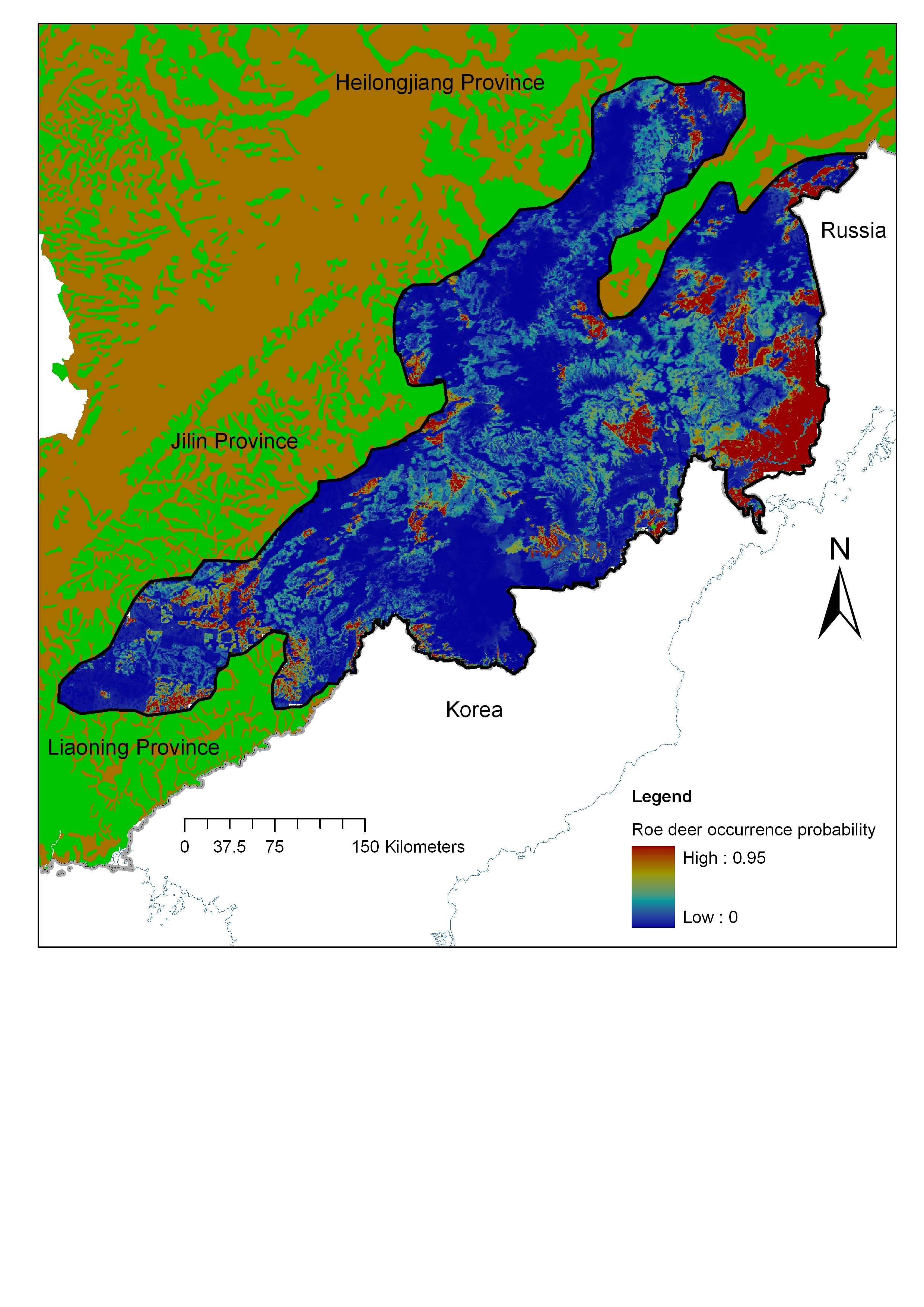

Supplement: Supplementary Figures [file srep15475-s2.zip › Extended data Fig. 8 Roe deer occurrence probability.jpg]

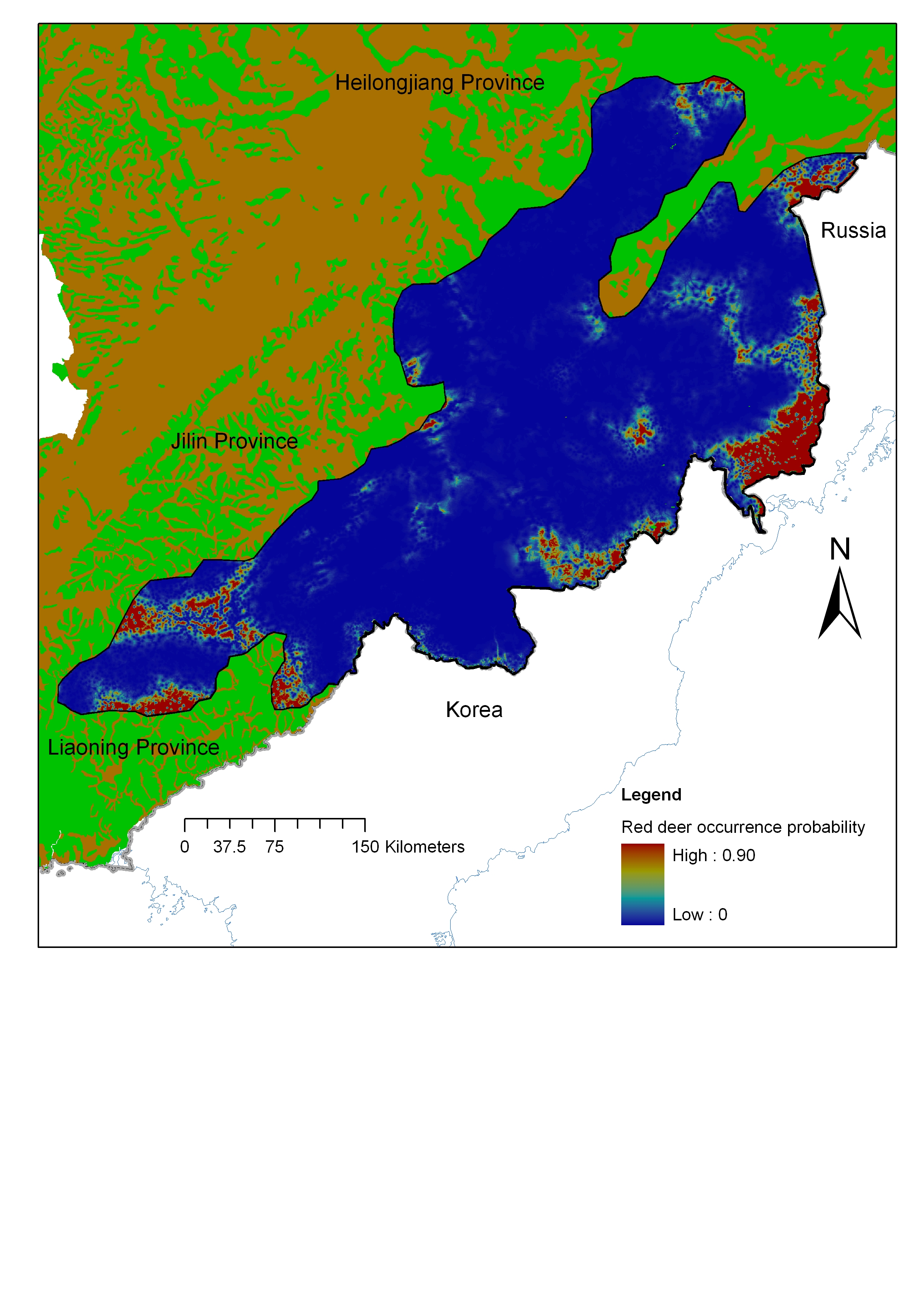

Supplement: Supplementary Figures [file srep15475-s2.zip › Extended data Fig. 9 Red deer occurrence probability.jpg]

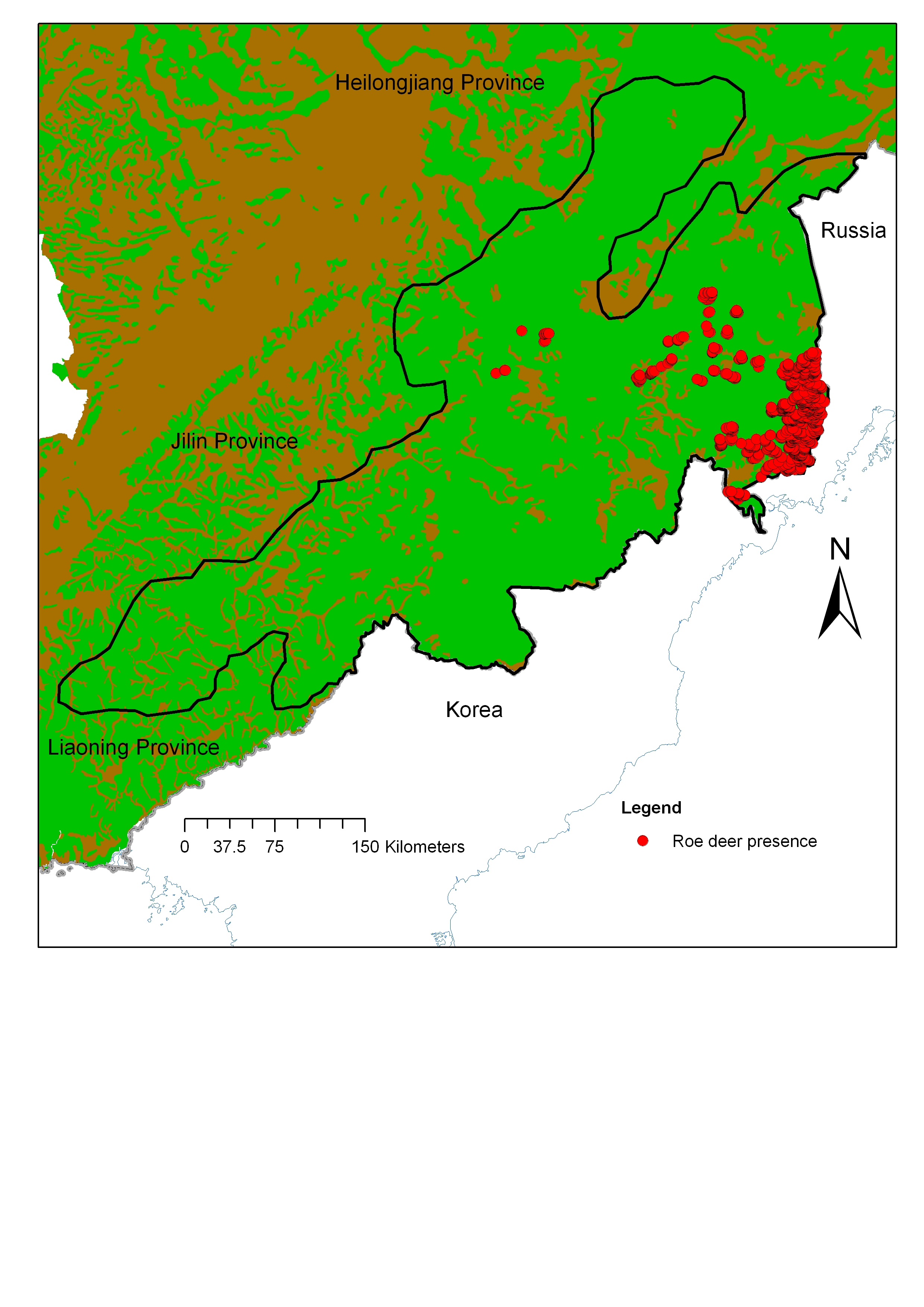

Supplement: Supplementary Figures [file srep15475-s2.zip › Extended data Fig.1 roe deer presence.jpg]

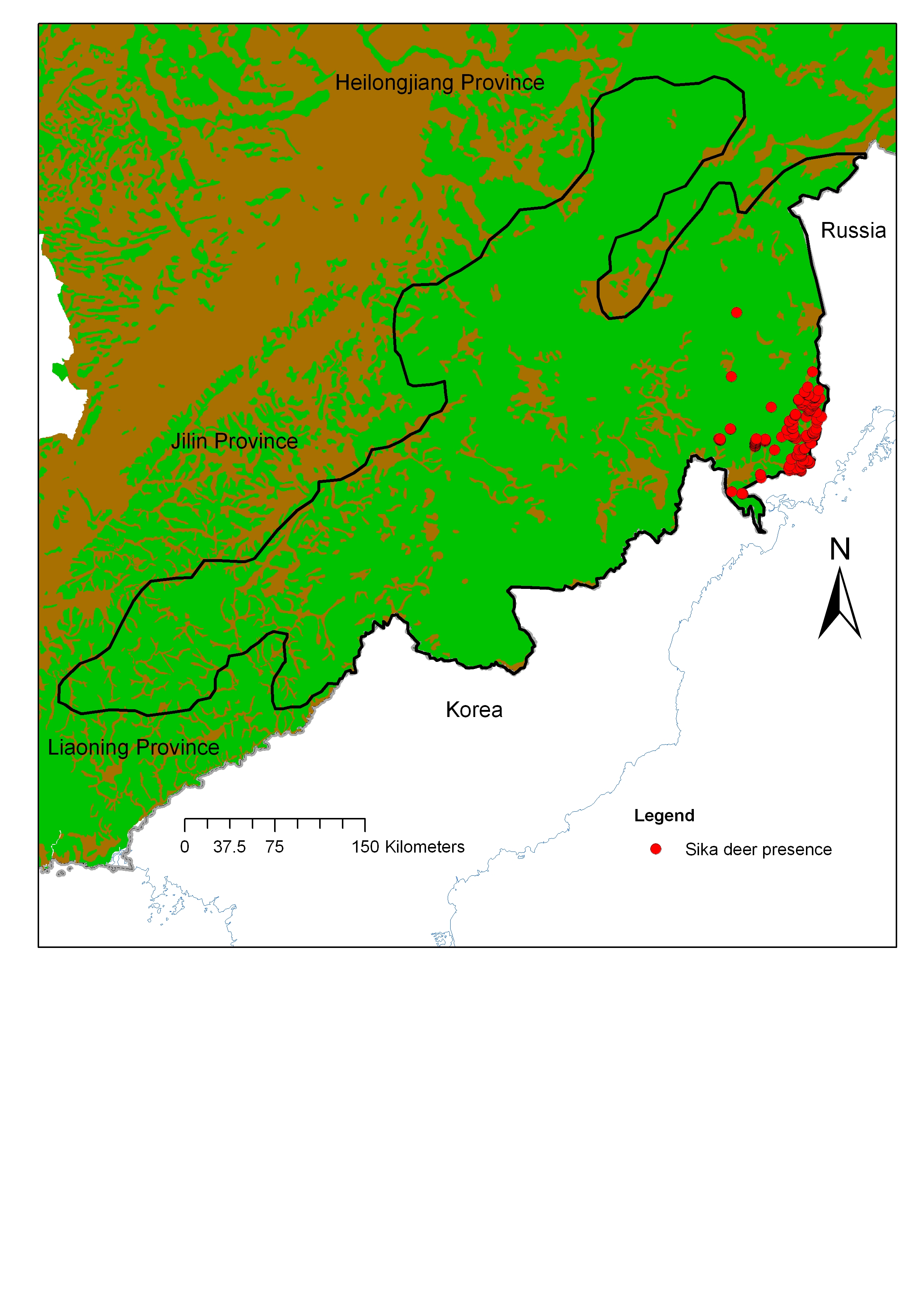

Supplement: Supplementary Figures [file srep15475-s2.zip › Extended data Fig.3 Sika deer presence.jpg]

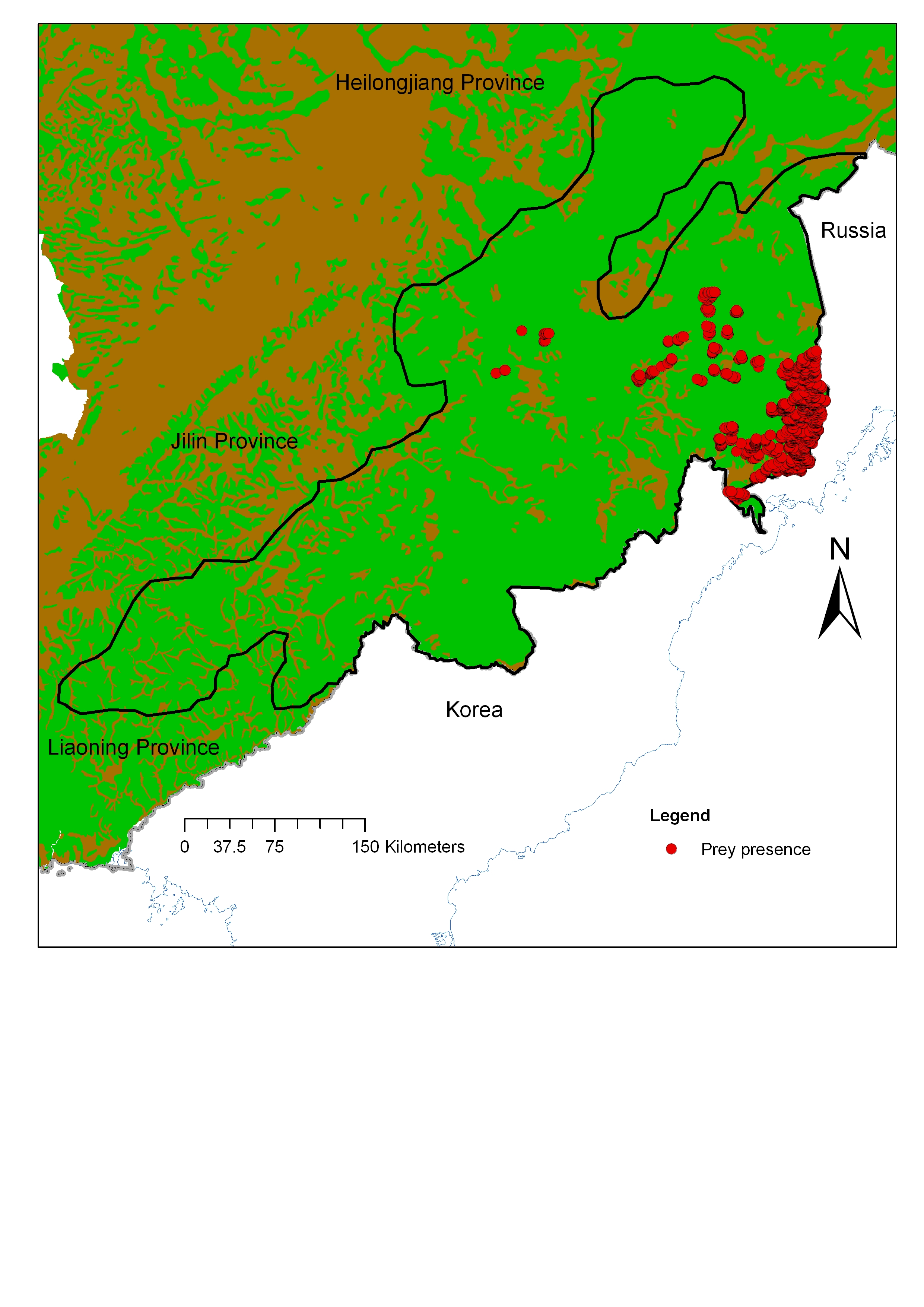

Supplement: Supplementary Figures [file srep15475-s2.zip › Extended data Fig.5 Prey presence.jpg]

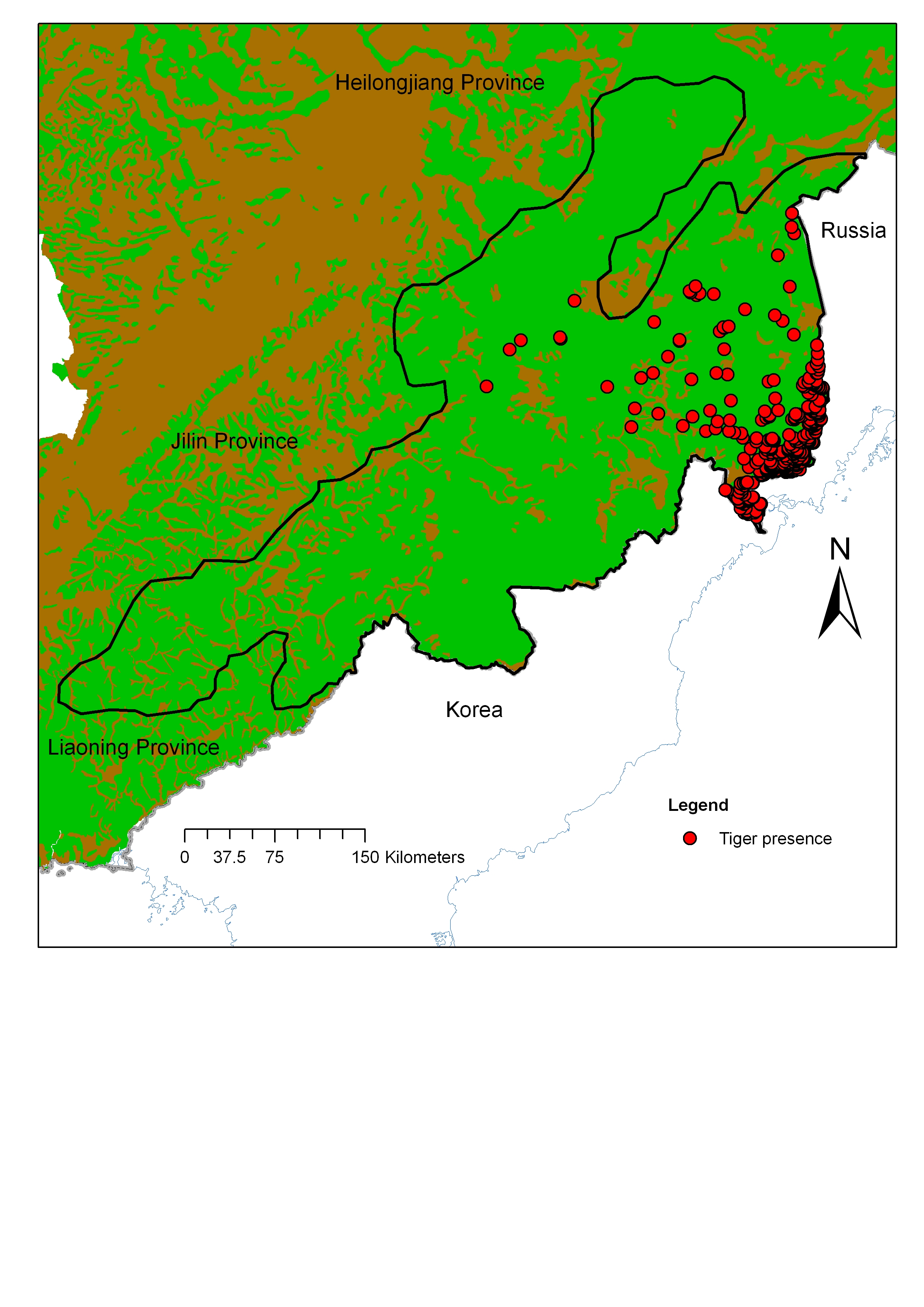

Supplement: Supplementary Figures [file srep15475-s2.zip › Extented data Fig. 6 Amur tiger presence.jpg]
