# Supplementary material for: New hope for the survival of the Amur leopard in China
Source: Sci Rep. 2015 Dec 7;5:15475. doi: 10.1038/srep15475 (PMC4670984; doi:10.1038/srep15475)
Supplement: Supplementary Figures [file srep15475-s2.zip › Extended data Fig. 14 Curve response to ungulate and Amur tiger.doc]

Roe deer

Red deer

Wild boar

Sika deer

Prey

Amur tiger
